# The physiological role of orexin/hypocretin neurons in the regulation of sleep/wakefulness and neuroendocrine functions

**DOI:** 10.3389/fendo.2013.00018

**Published:** 2013-03-06

**Authors:** Ayumu Inutsuka, Akihiro Yamanaka

**Affiliations:** Department of Neuroscience II, Research Institute of Environmental MedicineNagoya University, Nagoya, Japan

**Keywords:** orexin, hypocretin, sleep, hypothalamus, optogenetics, neuropeptide

## Abstract

The hypothalamus monitors body homeostasis and regulates various behaviors such as feeding, thermogenesis, and sleeping. Orexins (also known as hypocretins) were identified as endogenous ligands for two orphan G-protein-coupled receptors in the lateral hypothalamic area. They were initially recognized as regulators of feeding behavior, but they are mainly regarded as key modulators of the sleep/wakefulness cycle. Orexins activate orexin neurons, monoaminergic and cholinergic neurons in the hypothalamus/brainstem regions, to maintain a long, consolidated awake period. Anatomical studies of neural projections from/to orexin neurons and phenotypic characterization of transgenic mice revealed various roles for orexin neurons in the coordination of emotion, energy homeostasis, reward system, and arousal. For example, orexin neurons are regulated by peripheral metabolic cues, including ghrelin, leptin, and glucose concentration. This suggests that they may provide a link between energy homeostasis and arousal states. A link between the limbic system and orexin neurons might be important for increasing vigilance during emotional stimuli. Orexins are also involved in reward systems and the mechanisms of drug addiction. These findings suggest that orexin neurons sense the outer and inner environment of the body and maintain the proper wakefulness level of animals for survival. This review discusses the mechanism by which orexins maintain sleep/wakefulness states and how this mechanism relates to other systems that regulate emotion, reward, and energy homeostasis.

## INTRODUCTION

The hypothalamus plays a critical role in maintaining energy homeostasis by coordinating behavioral, metabolic, and neuroendocrine responses (Bernardis and Bellinger, 1996). Within this region, the lateral hypothalamic area (LHA) has been regarded as an important center for feeding and arousal because animal models with LHA lesions exhibit hypophagia and decreased arousal that frequently leads to death. Orexin A and orexin B (also known as hypocretin 1 and hypocretin 2) are neuropeptides expressed exclusively by LHA neurons. Orexin-producing neurons (orexin neurons) project their axons throughout the brain (Peyron et al., 1998; Nambu et al., 1999), which suggests that their functions are varied. Remarkably, dense projections of orexin neurons are observed in the serotonergic dorsal raphe nucleus (DR), noradrenergic locus coeruleus (LC), and histaminergic tuberomammillary nucleus (TMN); and all of these nuclei are involved in promoting arousal (Saper et al., 2005). Prepro-orexin knockout mice, orexin receptor knockout mice, and orexin neuron-ablated transgenic mice all show severely defective sleep/wakefulness cycles (Chemelli et al., 1999; Hara et al., 2001; Willie et al., 2003). Consistently, deficiencies of orexin function were found in human narcolepsy (Nishino et al., 2000; Peyron et al., 2000; Thannickal et al., 2000). These findings clearly show the importance of the orexin system in the regulation of sleep/wakefulness. Past studies also revealed roles for orexin neurons beyond feeding and arousal, including autonomic nervous system control (Sellayah et al., 2011; Tupone et al., 2011) and in reward and stress systems (Boutrel et al., 2005; Harris et al., 2005).

In this review, we first discuss the basic biological features of orexins and their receptors, and we then describe the neuronal inputs and outputs of the orexin neurons. Finally, we discuss the various physiological roles of the orexin system, focusing on the regulation of sleep and wakefulness.

## OREXIN AND OREXIN RECEPTORS

In 1998, two groups independently found the same new peptides by using different strategies. Sakurai et al. (1998) used reverse pharmacology to identify ligands of orphan G-protein-coupled receptors (GPCRs). They found a novel family of neuropeptides that binds to two closely related orphan GPCRs. Because the injection of the ligands induced feeding behavior, they named the ligands “orexin” after the Greek word orexis, which means appetite (Sakurai et al., 1998). At the same time, de Lecea et al. (1998) isolated cDNAs selectively expressed within the hypothalamus. Two peptides of the cDNAs showed substantial amino acid sequence homology with the gut peptide hormone secretin, so they named these peptides “hypocretin.” They suggested that hypocretins function within the central nervous system as neurotransmitters.

Prepro-orexin polypeptide is proteolysed to produce two orexins, orexin A and orexin B. Orexin A is a 33-amino acid peptide of 3.5 kDa, with an N-terminal pyroglutamyl residue and C-terminal amidation. The four Cys residues of orexin A form two sets of intrachain disulfide bonds. This structure is completely conserved among several mammalian species (human, rat, mouse, cow, sheep, dog, and pig). On the other hand, rat orexin B is a 28-amino acid, C-terminally amidated, linear peptide of 2.9 kDa, which is 46% identical in sequence to rat orexin A. The 3.2 kb fragment of the 5′-upstream region of the human prepro-orexin gene is reported to be sufficient to express genes in orexin-containing neurons (Sakurai et al., 1999; Moriguchi et al., 2002).

*In situ* hybridization of prepro-orexin shows orexin-containing neurons are located in the LHA. Prepro-orexin mRNA was shown to be upregulated under fasting conditions, indicating that these neurons somehow sense the animal's energy balance (Sakurai et al., 1998). Recently, the forkhead box transcription factor Foxa2, a downstream target of insulin signaling, was reported to be involved in this transcriptional regulation (Silva et al., 2009).

**FIGURE 1:**
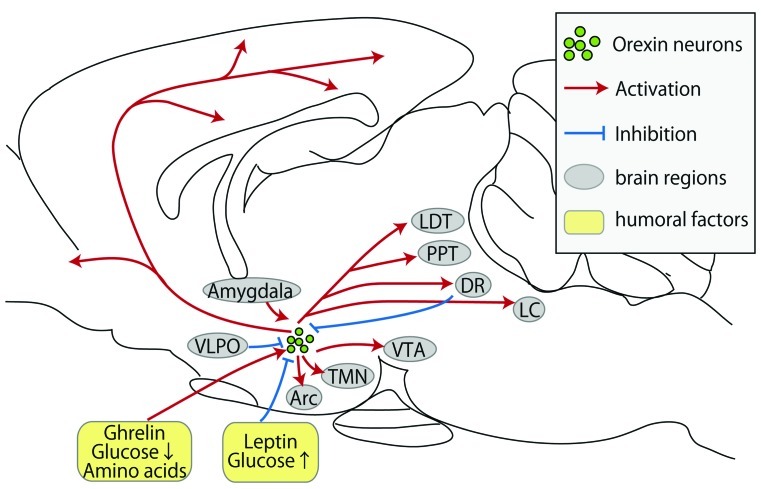
**Schematic representation of inputs and outputs of orexin neurons.** Orexin neurons are found only in the lateral hypothalamic area but project throughout the entire central nervous system. Red arrows show excitatory projections, while blue lines show inhibitory projections. Abbreviations: Arc, arcuate nucleus; DR, dorsal raphe nucleus; LC, locus coeruleus; LDT, laterodorsal tegmental nucleus; PPT, pedunculopontine tegmental nucleus; TMN, tuberomammillary nucleus; VTA, ventral tegmental area; VLPO, ventrolateral preoptic nucleus.

Orexin A acts on both orexin receptor 1 (OX1R) and 2 (OX2R), while orexin B selectively acts on OX2R (Sakurai et al., 1998). While orexin neurons are localized within the LHA, they have widespread projections throughout the brain (Peyron et al., 1998; Nambu et al., 1999; **Figure 1**). Therefore, it is important to know the distribution pattern of orexin receptors to identify the functional neuronal network. Marcus et al. (2001) used *in situ* hybridization to demonstrate that OX1R and OX2R differ in distribution. OX1R mRNA was observed in many brain regions including hippocampus, paraventricular thalamic nucleus (PVN), ventromedial hypothalamic nucleus, DR, and LC. OX2R mRNA was prominent in a complementary distribution including the cerebral cortex, hippocampus, DR, and many hypothalamic nuclei including PVN, TMN, and the ventral premammillary nucleus. Among these regions, DR, LC, and TMN are well known to be involved in maintenance of the awake state. Consistently, orexin-deficient mice display a narcolepsy-like phenotype (Chemelli et al., 1999), as do dogs with a mutation preventing the expression of OX2R (Lin et al., 1999). Note that the regions expressing orexin receptors contain several areas of the hypothalamus, including LHA, PVN, and the arcuate nucleus (Arc), which are all strongly implicated in the modulation of feeding.

## SIGNAL TRANSDUCTION SYSTEM OF OREXIN NEURONS

OX1R and OX2R are seven-transmembrane GPCRs, which transmit information into the cell by activating heterotrimeric G proteins. The signal transduction pathways of orexin receptors were examined in cells transfected with OX1R or OX2R. The inhibitory effect of orexin on forskolin-stimulated cyclic adenosine monophosphate (cAMP) accumulation was not observed in OX1R-expressing cells. In addition, orexin-stimulated elevation in [Ca^2^^+^]i in OX1R- or OX2R-expressing cells was not affected by pertussis toxin (PTX) pretreatment. These results suggest that OX1R does not couple to Gi proteins. On the other hand, forskolin-stimulated cAMP accumulation in OX2R-expressing cells was inhibited by orexin in a dose-dependent manner, and this effect was abolished by pretreatment with PTX. These results indicate that OX2R couples to both PTX-sensitive and PTX-insensitive proteins (Zhu et al., 2003). Note that orexin has two independent actions on neuronal activity: activation of noisy cation channels that generate depolarization and activation of a protein kinase C (PKC)-dependent enhancement of Ca^2^^+^ transients mediated by L-type Ca^2^^+^ channels (Kohlmeier et al., 2008).

Orexin neurons innervate monoaminergic neurons. In particular, noradrenergic neurons of the LC, dopaminergic neurons of the ventral tegmental area (VTA), and histaminergic neurons of the TMN are activated by orexins (Hagan et al., 1999; Horvath et al., 1999b; Nakamura et al., 2000; Yamanaka et al., 2002). LC neurons exclusively express OX1R, while TMN neurons express OX2R, suggesting that both OX1R and OX2R signaling are excitatory on neurons. Orexin colocalizes with dynorphin (Chou et al., 2001) and glutamate (Abrahamson et al., 2001). It has also been demonstrated that orexin increases local glutamate signaling by facilitation of glutamate release from presynaptic terminals (Li et al., 2002).

## INPUT TO OREXIN NEURONS

### ANATOMICAL ANALYSIS OF NEURONAL INPUT TO OREXIN NEURONS

It has been challenging to study the neuronal afferents to orexin neurons because they are scattered sparsely within the LHA. To address this point, retrograde tracing studies were performed. The non-toxic C-terminal fragment of tetanus toxin (TTC) can be utilized to retrogradely transfer the fused protein to interconnected neurons and transport toward the cell bodies of higher-order neurons (Maskos et al., 2002). Sakurai et al. (2005) generated transgenic mouse lines expressing a fused protein of TTC and green fluorescent protein (GFP) exclusively in orexin neurons by using the promoter of human prepro-orexin. They identified several brain regions including the basal forebrain cholinergic neurons, gamma-aminobutyric acid (GABA)ergic neurons in the ventrolateral preoptic nucleus (VLPO), and serotonergic neurons in the median raphe and paramedian raphe nucleus. Moreover, regions associated with emotion including the amygdala, infralimbic cortex, shell region of the nucleus accumbens, and the bed nucleus of the stria terminalis (BST) were found to innervate orexin neurons.

In addition to TTC, the cholera toxin B subunit (CTB) is also used to retrogradely trace neuronal projections. Yoshida et al. (2006) injected CTB into the LHA and counted every labeled cell in rats. Interestingly, they found strong projections from the lateral septum, preoptic area, BST, and posterior hypothalamus. In addition, they also found that hypothalamic regions preferentially innervate orexin neurons in the medial and perifornical parts of the field, but most projections from the brainstem target the lateral part of the field.

The results of these two papers present slight distinctions. TTC::GFP sometimes labeled regions with no known projections to the orexin field such as the medial septum possibly because of transport to second-order neurons or ectopic expression of the transgene. In addition, the TTC::GFP technique also appears to be less sensitive than conventional retrograde tracers, as it failed to label neurons in the lateral septum or VTA – regions that probably innervate orexin neurons as indicated by anterograde tracing and other retrograde tracing studies (Yoshida et al., 2006; Richardson and Aston-Jones, 2012).

Given that inputs to orexin neurons are so anatomically varied and associated with multiple functions, it might be reasonable to hypothesize the existence of subgroups of orexin neurons. Indeed, anterograde tracers injected into the DR marked the lateral LHA preferentially, while injections into the VMH preferentially stained neurons in the medial LHA (Yoshida et al., 2006). With current technology we cannot only trace neuronal projections but also analyze functional connectivity by utilizing optogenetic and pharmacogenetic tools (Lammel et al., 2012).

### INPUT FROM HYPOTHALAMUS

Previous studies indicated that the LHA is innervated by several hypothalamic regions, and some of these innervations project toward orexin neurons in the LHA. The LHA has long been considered a brain region regulating food intake and body weight. The localization of orexin neurons to the LHA and the functions ascribed to orexin neurons suggest that they may constitute components of a central circuitry controlling energy metabolism. Therefore, it is reasonable to assess their connectivity to other neuronal populations involved in ingestive behaviors. Neuropeptide Y (NPY), produced by specific neurons in the hypothalamic Arc (Allen et al., 1983; Chronwall et al., 1985), was demonstrated to be a prominent inducer of food intake upon central administration (Clark et al., 1984; Stanley and Leibowitz, 1985). Projections to orexin neurons from NPY/agouti-related peptide (AgRP) neurons in the Arc were identified by anatomical studies (Broberger et al., 1998; Elias et al., 1998). Furthermore, orexin neurons express NPY receptors, and direct administration of NPY agonists into the LHA increases Fos-like immunoreactivity in orexin neurons (Campbell et al., 2003). These findings suggest NPY excites orexin neurons; however, electrophysiological analyses showed that direct application of NPY instead reduces spike frequency and hyperpolarizes the membrane potential of orexin neurons (Fu et al., 2004).

### INPUT FROM LIMBIC SYSTEM

Given that depletion of orexin neurons induces the sleep disorder narcolepsy, the limbic system might also provide important projections to orexin neurons. Narcolepsy patients often suffer from an attack called “cataplexy,” which is characterized by sudden weakening of postural muscle tone. Cataplexy is often triggered by strong, generally positive emotion while consciousness is preserved during the attack (Honda et al., 1986). This fact implies that orexin neurons may play a role in the physiological responses associated with emotions. Consistently, local injection of orexin into the pedunculopontine tegmental nucleus (PPT) strongly inhibited rapid eye movement (REM)-related atonia in the cat (Takakusaki et al., 2005). Therefore, it is hypothesized that emotional stimuli increase orexin release in the PPT to prevent muscle atonia in wild-type animals.

The innervations from limbic system may mediate emotional arousal and fear-related responses. Prepro-orexin knockout mice showed weaker cardiovascular and locomotor responses to emotional stress in an awake and freely moving condition (Kayaba et al., 2003). Consistently, air jet stress-induced elevations of blood pressure and heart rate were attenuated in conscious orexin/ataxin-3 mice, in which orexin neurons were specifically ablated by expressing neurotoxic protein (Zhang et al., 2006).

### INPUT FROM PREOPTIC AREAS

The preoptic area, especially the VLPO, plays a critical role in non-REM sleep initiation and maintenance (Lu et al., 2002). The VLPO has multiple inhibitory projections to neurons that release wake-promoting neurotransmitters, including histamine neurons in the TMN, noradrenergic neurons in the LC, serotonergic neurons in the DR, and acetylcholinergic neurons (Sherin et al., 1996, 1998; Steininger et al., 2001; Lu et al., 2002).

Neurons in the VLPO fire at a rapid rate during sleep, with attenuation of firing during the awake period. Likewise, neurons in wake-promoting centers fire rapidly during wakefulness and are relatively quiescent during sleep, with the exception of cholinergic neurons, which are divided into two classes of neurons: one is active in both the awake and REM sleep period, and the other is active only in the REM sleep period.

Orexin neurons are strongly inhibited by both a GABA_A_ agonist, muscimol (Yamanaka et al., 2003b), and a GABA_B_ receptor agonist, baclofen (Xie et al., 2006). Orexin neurons are also innervated by cells in the VLPO that also contain GABA (Sakurai et al., 2005; Yoshida et al., 2006). These observations suggest that VLPO neurons send GABAergic inhibitory projections to wake-promoting neurons including orexin neurons. This pathway might be important to initiate and maintain sleep.

### INPUT FROM SUPRACHIASMATIC NUCLEUS

Given that sleep/wakefulness is a circadian phenomenon, it is reasonable to consider that orexin neurons receive information from the suprachiasmatic nucleus (SCN), which is the center of the circadian rhythm according the environmental light–dark information. Indeed, the circadian fluctuation of orexin levels in the cerebrospinal fluid (CSF) disappears when the SCN is removed (Deboer et al., 2004). Although direct input to orexin neurons from the SCN appears to be sparse, orexin neurons receive abundant innervations from the BST, supraventricular zone, and dorsomedial hypothalamus (DMH; Sakurai et al., 2005; Yoshida et al., 2006), which receive input from the SCN (Leak and Moore, 2001). This suggests the possibility that orexin neurons receive circadian influences from the SCN via these regions. Note that excitotoxic lesions of the DMH reduce the circadian rhythmicity of wakefulness. The DMH projects to orexin neurons (Chou et al., 2003), although the DMH also projects to multiple brain areas such as the LC and the VLPO that are involved in sleep and wakefulness. In addition, considering that the orexin system is involved in food-entrainable oscillator (FEO; Mieda et al., 2004), the circadian change in the activity of orexin neurons might be regulated by other elements, such as energy balance.

## FACTORS THAT INFLUENCE ACTIVITY OF OREXIN NEURONS

Electrophysiological studies have identified several modulators that regulate activity of orexin neurons. Recordings from transgenic mice expressing GFP in orexin neurons demonstrated that agonists of ionotropic glutamate receptors activated orexin neurons, while glutamate antagonists reduced their activity (Li et al., 2002; Yamanaka et al., 2003b). These results indicate that orexin neurons are tonically activated by glutamate.

Dopamine, noradrenaline, and serotonin (5-HT) hyperpolarize and inhibit orexin neurons via alpha2 and 5-HT_1A_ receptors, respectively (Yamanaka et al., 2003b; Muraki et al., 2004; Li and van den Pol, 2005). Dopamine-induced hyperpolarization is most likely mediated by alpha2-adrenergic receptors since a very high concentration of dopamine is necessary to induce hyperpolarization and also because dopamine-induced hyperpolarization is inhibited by the alpha2-adrenergic receptor antagonist, idazoxan (Yamanaka et al., 2006). However, it is noteworthy that dopamine potentially affects both dopamine receptors and adrenergic receptors, while the dopamine D2 receptor antagonist eticlopride blocks the actions of dopamine on spike frequency and membrane potential (Li and van den Pol, 2005). Thus, dopamine might act through both alpha2-adrenergic receptor and dopamine D2 receptor.

Recently, it was found that orexin itself excites orexin neurons via OX2R (Yamanaka et al., 2010). This suggests that orexin neurons form a positive-feedback circuit through indirect and direct pathways, which results in the preservation of the orexin neuron network at a high activity level and/or for a longer period.

Calcium imaging using transgenic mice in which orexin neurons specifically express yellow cameleon 2.1 showed that neurotensin, sulfated octapeptide form of cholecystokinin, oxytocin, and vasopressin activate orexin neurons, while 5-HT, noradrenaline, dopamine, and muscimol, a GABA_A_ receptor agonist inhibit these cells (Tsujino et al., 2005). Recently, it was also reported that orexin neurons express glycine receptors throughout adulthood and that glycine inhibits the electric activity of orexin neurons directly and indirectly (Hondo et al., 2011; Karnani et al., 2011b).

Other factors that reportedly influence the activity of orexin neurons include corticotrophin-releasing factor (Winsky-Sommerer et al., 2004), ATP (Wollmann et al., 2005), NPY (Fu et al., 2004), and physiological fluctuations in acid and CO_2_ levels (Williams et al., 2007). It is noteworthy that the factors that are supposed to be influenced by feeding (such as glucose, ghrelin, and leptin) inhibit the activity of orexin neurons (Yamanaka et al., 2003a). The large variety of factors regulating orexin neuronal activities demonstrates the integral role of orexin neurons in monitoring circadian rhythms, energy balance, and vigilance level.

## REGULATION OF OREXIN NEURONS BY HUMORAL FACTORS

Motivated behaviors such as food-seeking are deeply involved in maintenance of arousal. Orexin neurons are thought to function as the sensor of energy balance. Electrophysiological studies revealed that increasing extracellular glucose concentrations induce striking hyperpolarizations, while decreasing the glucose concentration induces depolarization and increases the frequency of action potentials of orexin neurons (Yamanaka et al., 2003a; Burdakov et al., 2005). Importantly, this mechanism is sufficiently sensitive to respond to physiological fluctuations of glucose concentration induced by normal feeding. Note that other dietary nutrients, amino acids, also activate orexin neurons, and they can suppress the glucose response of orexin neurons at physiological concentration (Karnani et al., 2011a).

In addition, the orexigenic peptide ghrelin activated isolated orexin neurons with depolarization and an increase in action potential frequency (Yamanaka et al., 2003a). In contrast, the strong anorectic factor leptin robustly inhibited orexin neurons, causing hyperpolarization and decreasing the firing rate (Yamanaka et al., 2003a). Notably, insulin exerted no direct effects on orexin neurons. These findings are consistent with the idea that orexin neurons act as a sensor of nutritional status (Sakurai et al., 1998). Indeed, transgenic mice without orexin neurons fail to show fasting-induced arousal (Yamanaka et al., 2003a).

## PHYSIOLOGICAL FUNCTIONS OF OREXIN NEURONS

### FUNCTIONS IN FEEDING BEHAVIORS AND ENERGY HOMEOSTASIS

Orexin neuron-ablated transgenic mice show hypophagia and late-onset obesity (Hara et al., 2001), although the severity of the obese phenotype critically depends on genetic background (Hara et al., 2005). These findings imply a role for orexin in the regulation of energy homeostasis. Although orexins stimulate feeding behavior, they do not slow metabolic rate, which might be expected in a system geared for weight gain. Instead, orexins increase both food intake and metabolic rate (Lubkin and Stricker-Krongrad, 1998). Because animals must be aware and active when they seek and eat food, this function might be important for feeding behavior.

The Arc is attributed to the regulation of feeding behaviors. Orexin neurons densely project to this region (Date et al., 1999; Horvath et al., 1999a). Principal components of the regulating system of feeding behaviors include antagonistic and complementary appetite-stimulating (orexigenic) and appetite-suppressing (anorectic) pathways: NPY/AgRP neurons and proopiomelanocortin (POMC) neurons. It was reported that intracerebroventricular (ICV) injection of orexin induced c-Fos expression in NPY neurons of the Arc (Yamanaka et al., 2000). Therefore, orexin-stimulated feeding may occur at least partly through NPY pathways. However, because NPY antagonist (which completely abolished NPY-induced feeding) only partially abolished orexin-induced feeding in rats, other pathways by which orexin induces feeding might exist. POMC neurons of the Arc are known to suppress appetite, and lack of POMC-derived peptides or electrical silencing of POMC neurons causes obesity. Orexin neurons might affect feeding behavior by inhibiting POMC-expressing neurons (Muroya et al., 2004). Indeed, orexin suppresses action potential firing and hyperpolarizes the membrane potential of POMC neurons in the Arc (Ma et al., 2007).

Intracerebroventricular injection of orexin induces water intake as well as food intake (Kunii et al., 1999). Additionally arginine-vasopressin, also known as antidiuretic hormone, activates orexin neurons via the V1a receptor (Tsunematsu et al., 2008). These results suggest a role for orexin neurons in fluid homeostasis too.

### FUNCTIONS IN SLEEP AND WAKEFULNESS

The roles of orexin neurons in the regulation of sleep and arousal have been reported repeatedly. ICV injection of orexin A or orexin B during the light period increased awake time and reciprocally decreased REM and non-REM sleep time (Hagan et al., 1999; Bourgin et al., 2000; Piper et al., 2000). Sleep fragmentation observed in orexin knockout mice (Chemelli et al., 1999), orexin receptor knockout mice (Willie et al., 2003), and orexin neuron-ablated transgenic mice (Hara et al., 2001) shows us the importance of their physiological functions. Narcolepsy is a sleep disorder characterized by primary disorganization of sleep/wakefulness cycles. It has also been reported that the number of orexin neurons is greatly reduced, and orexin peptide levels in the cerebrospinal fluid are decreased to undetectable levels in narcoleptic patients (Nishino et al., 2000; Peyron et al., 2000; Thannickal et al., 2000). Orexin-ataxin-3 mice are a well known mouse model of narcolepsy. However, in orexin-ataxin-3 mice, orexin neurons are absent from birth, and therefore other neuronal mechanisms might compensate for the function of orexin neurons during development. Indeed, the frequency of cataplexy is not high in these mice. Timing-controlled neuronal ablation models using the tTA-TetO system might overcome this problem.

The activities of monoaminergic neurons in the brainstem and hypothalamus are known to be associated with sleep and awake states. Furthermore, the DR, LC, and TMN monoaminergic neurons express orexin receptors and are densely innervated by orexin neurons. These findings suggest that these regions mediate the effects of orexins. Consistently, noradrenergic neurons of the LC (Hagan et al., 1999), serotonergic neurons of the DR (Brown et al., 2002; Liu et al., 2002), and histaminergic neurons of the TMN (Huang et al., 2001; Yamanaka et al., 2002) have been shown to be activated by orexins. These observations suggest that the activity of these monoaminergic neurons is at least partly regulated by orexins. Orexins also have a strong direct excitatory effect on cholinergic neurons of the basal forebrain (Eggermann et al., 2001), which is hypothesized to play an important role in arousal.

The PPT and the laterodorsal tegmental nucleus (LDT) provide cholinergic afferents to several brain regions and play a pivotal role in the regulation of REM sleep and wakefulness. These regions are also strongly innervated by orexin neurons (Peyron et al., 1998; Nambu et al., 1999). Electrophysiological experiments revealed that the firing rate of cholinergic neurons is increased by orexin A (Burlet et al., 2002). Microinjection of orexin A into the LDT increases awake time and decreases REM sleep time in cats (Xi et al., 2001), and when orexin A is injected into the PPT in cats, an increased stimulus at the PPT is required to induce muscle atonia (Takakusaki et al., 2005). However, it is noteworthy that the LDT/PPT contains other neuronal types beside cholinergic neurons that show activity associated with sleep/wake cycles (Sakai, 2012).

Emerging new studies using optogenetics have revealed the physiological roles of orexin neurons *in vivo*. Direct selective photostimulation of orexin neurons expressing channelrhodopsin2 increases the probability of transition from non-REM or REM sleep to wakefulness (Adamantidis et al., 2007) and activates downstream wake-promoting nuclei such as LC and TMN (Carter et al., 2009). Consistently, it was also shown that direct selective inhibition of orexin neurons expressing halorhodopsin induces non-REM sleep (Tsunematsu et al., 2011). Furthermore, optogenetic stimulation of the LC produces immediate sleep-to-wake transitions, whereas the inhibition causes a decrease in wakefulness (Carter et al., 2010). Recently it was also reported that photoinhibition of LC neurons during the photostimulation of orexin neurons cancels these sleep-to-wake transitions (Carter et al., 2012). These findings indicate that the LC is a major effector of orexin neurons in the regulation of sleep and wakefulness. However, it is noteworthy that LC noradrenergic neurons express only OX1R, and that OX1R knockout mice show only weak fragmentation of sleep and no cataplexy (Mieda et al., 2011).

Recently, not only photo-activated ion channels or ion pumps but also other natural and modified proteins have been used to regulate the activity of specific neuronal circuits *in vivo*. One interesting example is “Designer Receptors Exclusively Activated by Designer Drugs (DREADD).” This method employs modified muscarinic receptors (hM3Dq for excitation and hM4Di for inhibition) that have lost their affinity for endogenous acetylcholine but can be activated by a synthetic ligand, clozapine-N-oxide, which can cross the blood–brain barrier (Armbruster et al., 2007; Alexander et al., 2009). Because stimulation of GPCRs with a specific ligand has a longer effect on cellular signaling than optical stimulation, the DREADD system can facilitate the examination of the chronic effects of modulating the activity of specific neurons. Using this technique, it was reported that the excitation of orexin neurons significantly increased the amount of time spent in wakefulness and decreased both non-REM and REM sleep times and that inhibition of orexin neurons decreased wakefulness time and increased non-REM sleep time (Sasaki et al., 2011). Additionally, melanopsin, a photosensitive G-protein-coupled photopigment, makes it possible to control wakefulness by blue light in a way similar to channelrhodopsin (Tsunematsu et al., 2012).

### FUNCTIONS IN AUTONOMIC NERVOUS SYSTEM

Orexin-deficient mice show lower blood pressure than wild-type littermates (Kayaba et al., 2003; Zhang et al., 2006). Consistently, ICV injection of orexins increases blood pressure and heart rate (Shirasaka et al., 1999), and these effects are abolished by administration of alpha1-adrenergic receptor antagonist, prazosin, or beta-adrenergic receptor antagonist, propranolol. These results suggest that orexins physiologically stimulate the sympathetic nervous system and regulate energy expenditure.

Heat production in brown adipose tissue (BAT) also contributes to body weight regulation through the maintenance of body temperature. Recently Tupone et al. (2011) reported that orexinergic projections to raphe pallidus increase BAT thermogenesis in rat. This finding provides a new mechanism contributing to the disrupted regulation of body temperature and energy metabolism in the absence of orexin. Orexin neuron-ablated transgenic mice show late-onset obesity, although they also show hypophagia (Hara et al., 2001). The regulation of BAT thermogenesis by orexin neurons might account for this phenotype of energy metabolism (Sellayah et al., 2011).

### FUNCTIONS IN REWARD AND STRESS SYSTEMS

To attenuate the symptoms of the sleep disorder, psychostimulants such as amphetamine or methylphenidate are often given to narcolepsy patients. Interestingly, drug addiction hardly occurs in these patients. This finding suggests that the orexin system mediates the establishment of drug addiction. The LHA, where orexin neurons exist, is a brain region historically implicated in reward and motivation, and orexin neurons project to many brain areas including the LC, nucleus accumbens, and VTA (Fadel and Deutch, 2002) that are implicated in behavioral responses to drugs of abuse. Orexin directly activates VTA dopaminergic neurons (Nakamura et al., 2000; Korotkova et al., 2003). ICV or local VTA infusion of orexin drives behavior motivated by either food or drug rewards (Sakurai et al., 1998; Boutrel et al., 2005; Harris et al., 2005).

It was demonstrated that orexin A input to the VTA potentiates *N*-methyl-D-aspartate receptor (NMDAR)-mediated neurotransmission via a PLC/PKC-dependent insertion of NMDARs in VTA dopamine neuron synapses (Borgland et al., 2006). Furthermore, intra-VTA microinjection of an OX1R antagonist abolished a conditioned place preference for morphine (Narita et al., 2006) and locomotor sensitization to cocaine (Borgland et al., 2006). These data indicate that orexin signaling plays an important role in neural plasticity relevant to addiction in the VTA.

## CONCLUDING REMARKS

**FIGURE 2:**
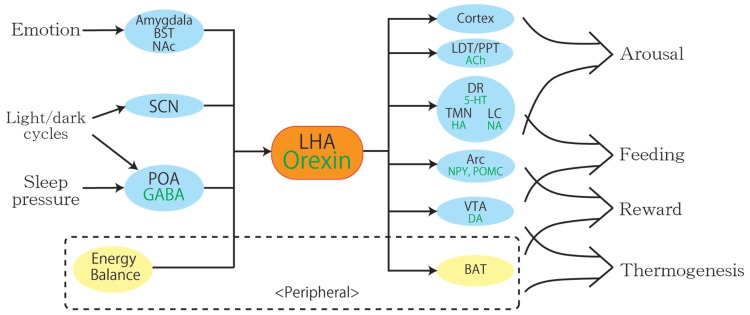
**A schematic diagram to illustrate the integrative physiological roles of orexin neurons.** Orexin neurons regulate various physiological phenomena such as wakefulness, feeding, reward, and thermogenesis. The body energy level influences orexin neuronal activity to coordinate arousal and energy homeostasis. Inputs from the limbic system may be important to regulate the activity of orexin neurons to evoke emotional arousal or fear-related responses. Abbreviations: 5-HT, serotonin; ACh, acetylcholine; Arc, arcuate nucleus; BAT, brown adipose tissue; BST, bed nucleus of the stria terminalis; DA, dopamine; DR, dorsal raphe nucleus; GABA, gamma-aminobutyric acid; HA, histamine; LC, locus coeruleus; LDT, laterodorsal tegmental nucleus; LHA, lateral hypothalamic area; NA, noradrenalin; NAc, nucleus accumbens; NPY, neuropeptide Y; POA, preoptic area; POMC, proopiomelanocortin; PPT, pedunculopontine tegmental nucleus; SCN, suprachiasmatic nucleus; TMN, tuberomammillary nucleus; VTA, ventral tegmental area.

Although the name orexin is derived from the word orexigenic after its function in feeding, mounting evidence has revealed various physiological roles for orexin other than feeding, such as maintenance of sleep, autonomous regulation, and reward processing. Orexin neurons in the LHA are anatomically well placed to provide a link between the limbic system, energy homeostasis, and brain stem monoaminergic or cholinergic neurons. Like the hypothalamus where orexin neurons exist, orexin neurons themselves monitor various physiological conditions and coordinate various behaviors to respond to environmental change adequately (**Figure 2**). For example, feeding behaviors affect the activity of orexin neurons through changes in concentration of glucose or amino acids, and these changes modulate the vigilance state, regulating aspects of the autonomic nervous system such as blood pressure, heart rate, and thermogenesis at the same time. These findings indicate a critical role for orexin neurons in the regulation of vigilance states, according to internal and external environments, for survival.

By combining viral-mediated tracing, electrophysiology, and optogenetic manipulations, it might be determined that there are several subpopulations of orexin neurons that project to different target areas. For example, the distribution pattern of orexin neurons appears to be divided into two groups: medial and lateral. Some of the input projections to orexin neurons demonstrate a preference between these two areas as well. With new tools to manipulate specific neuronal projections, we can now study physiological differences within the orexin system. These upcoming findings may reveal that discrete functional units underlie the integral role of the orexin system.

## Conflict of Interest Statement

The authors declare that the research was conducted in the absence of any commercial or financial relationships that could be construed as a potential conflict of interest.

## Abbreviations

AgRP: agouti-related peptide
Arc: arcuate nucleus
BAT: brown adipose tissue
BST: bed nucleus of the stria terminalis
CTB: cholera toxin B subunit
DR: dorsal raphe nucleus
DREADD: designer receptors exclusively activated by designer drugs
FEO: food-entrainable oscillator
GPCRs: G-protein-coupled receptors
ICV: intracerebroventricular
LC: locus coeruleus
LDT: laterodorsal tegmental nucleus
LHA: lateral hypothalamic area
NMDAR: *N*-methyl-D-aspartate receptor
NPY: neuropeptide Y
OX1R: orexin receptor 1
OX2R: orexin receptor 2
PPT: pedunculopontine tegmental nucleus
PTX: pertussis toxin
PVN: paraventricular thalamic nucleus
SCN: suprachiasmatic nucleus
TMN: tuberomammillary nucleus
TTC: C-terminal fragment of tetanus toxin
VLPO: ventrolateral preoptic nucleus
VTA: ventral tegmental area.
